# Measles epidemic in pediatric population in Greece during 2017–2018: Epidemiological, clinical characteristics and outcomes

**DOI:** 10.1371/journal.pone.0245512

**Published:** 2021-01-20

**Authors:** Maria Gianniki, Tania Siahanidou, Evanthia Botsa, Athanasios Michos

**Affiliations:** First Department of Pediatrics, National and Kapodistrian University of Athens, “Aghia Sophia” Children’s Hospital, Athens, Greece; Laboratoire National de Santé, LUXEMBOURG

## Abstract

**Background and aim:**

A measles outbreak occurred in Greece during 2017–2018 affecting mainly pediatric population. The aim of the study was to describe the epidemiological and clinical characteristics of the cases diagnosed in the major pediatric tertiary hospital of Athens, where 26.5% of national pediatric measles cases were diagnosed and treated.

**Methods:**

This is a retrospective study of children 0–16 years old, who presented at the emergency department and/or were hospitalized with clinical presentation compatible with measles and diagnosis was confirmed with molecular detection of the measles RNA in pharyngeal swabs. Epidemiological, clinical and laboratory characteristics were retrieved from medical records and analyzed.

**Results:**

A total of 578 children with measles were identified during the study period. 322 (55.7%) were male with median age 36 months (range:1–193), while the largest number of documented cases (251; 43.4%) were children aged 1–5 years. Most children (429/578; 74.2%) belonged to the Roma minority and only 64 (11.1%) had Greek origin. 497 (91.5%) children were unvaccinated and 37 (6.8%) were partially vaccinated with measles vaccine. Hospitalization was required for 342 (59.2%) children, whereas one or more complications were reported in 230 (67.2%) of them. Most frequent complications were elevated transaminases (139; 40.6%), acute otitis media (72; 21%), dehydration (67; 19.6%) and pneumonia (58; 16.9%). 11 children (3.2%) required intensive care admission for altered mental status/status epilepticus (3), sepsis (2) and ARDS (6). 119/342 (34.8%) children were treated with antibiotics because of possible or confirmed bacterial coinfection. One death was reported, concerning an 11-month-old unvaccinated infant, with underlying dystrophy, who died of sepsis.

**Conclusion:**

Measles is not an innocent viral infection, as it is still characterized by high morbidity and complications rates. Unvaccinated or partially vaccinated populations could trigger new outbreaks, resulting in significant cost in public health. To avoid future measles outbreaks, high vaccination coverage should be achieved, as well as closing immunity gaps in the population and ensuring high-quality measles surveillance.

## Introduction

Measles is still an important public health problem worldwide causing significant morbidity and mortality. Measles can be serious, being associated with many complications and hospital admissions. According to World Health Organization (WHO), more than 140.000 people died from measles in 2018—mostly children under the age of 5 years, although it is a vaccine-preventable disease and can be prevented by immunization program strategies guided by laboratory-supported surveillance [1, 2].

Many countries in the World Health Organization European Region (EUR) have made substantial progress toward measles elimination. By end of 2017, 37 (70%) EUR countries had sustained interruption of measles transmission for ≥36 months and were verified to have eliminated endemic measles [3]. However, progress has been uneven between and within countries, leaving clusters of susceptible individuals unprotected, and resulting in periodic epidemics. During 2017–2018, a resurgence of measles occurred in EUR, with large-scale outbreaks in several countries that had achieved elimination [3].

Measles is a notifiable disease in Greece and surveillance is performed through the mandatory notification system using the European Commission’s case definition adapted in 2012 [4]. Throughout the last decade, the incidence of measles in Greece has presented a constant decline with sporadic clusters or outbreaks. The last outbreak occurred in 2010–2011 and since then only sporadic measles cases were notified [5]. Endemic transmission remained interrupted for a period of 36 months in 2014–2016, and thus, Greece was declared to have achieved elimination [6].

Nevertheless, a new outbreak occurred in Greece during 2017–2018, with a total of 3.258 cases. 2174 (66.7%) of them were children <16 years old, most of them in Southern Greece [7]. This resurgence of measles is connected to clusters of under-vaccinated populations, and specifically to the -hard to reach- minority of Roma. It is difficult to monitor uptake of measles-mumps-rubella vaccine among the Roma, but according to latest data, it is estimated to be low [4]. Roma communities have particularly been found to experience language and literacy, and discrimination, as barriers to vaccination and health service access [8]. As a result the WHO identified surveillance to be more effective at highlighting susceptible populations, as long as research to diagnose barriers for vaccination in these groups [9].

The scope of the present study was to describe the epidemiological, clinical characteristics and the outcomes of children diagnosed with measles in the major Tertiary Pediatric Hospital of Athens, Greece where almost 26% of total national pediatric cases were diagnosed and/or hospitalized [7].

## Patients and methods

This is a retrospective study of children 0–16 years old who were diagnosed with measles at "Aghia Sofia" Children's Hospital in Athens, between 1st of August 2017 to 30th of October 2018. This is a 750-bed tertiary hospital serving almost 40% of pediatric population in Athens metropolitan area. We included all the children who presented at the emergency department and/or were hospitalized with clinical presentation compatible with measles and diagnosis was confirmed with molecular detection of the measles RNA in pharyngeal swabs. Exclusion criteria included lack of molecular confirmation of measles, irrelevant of exposure history or clinical presentation. The protocol of the study was approved by the Scientific and Bioethics committee of the Hospital (N.23455/05-10-18).

Measles cases initially were identified from the mandatory measles notification forms from the hospital-based database which was not anonymized. Because this was a retrospective study there as not requirement for informed consent according to the hospital regulations and the local laws.

Medical records of the laboratory confirmed cases were reviewed for epidemiological, demographic data (age, sex, origin, vaccination status, area of residence), clinical, laboratory data and way of measles exposure (family contact, community, hospitalization).

As far as for immunization status, children were categorized into four groups: zero doses (unvaccinated), one dose (partial vaccination), two doses (fully vaccinated) or unknown vaccination status. Information on vaccination status was mainly obtained from patient vaccination booklets, considered as the best possible source, or in some cases, mostly in the Roma subpopulation group, by self-reporting. In the latter case, the accuracy of the data could not be checked.

In children who were hospitalized clinical characteristics (signs, symptoms, duration of hospitalization, complications, outcomes), laboratory data (Complete blood count, C-reactive protein, electrolytes, liver and renal function enzymes) and therapeutic interventions (antibiotics, vitamin A, immunoglobulin) were retrieved and analyzed.

## Statistical analysis

The Statistical analysis was performed using statistical package for the social sciences (SPSS, version 17; SPSS Inc., Chicago, Illinois, USA). Most of the data were expressed as mean ± SD or frequencies. Continuous variables were compared through Student’s t-test and proportions were compared by Chi-square test. Results were presented as mean ± SD and *P-value* less than 0.05 was considered statistically significant.

## Results

During the study period, 578 children with molecularly confirmed measles were identified which represent 26.5% (578/2174) of all children< 16 years old reported to the National Health Organization measles database. Among the 578 children, 322 (55.7%) were male and 256 (44.3%) were female. Median age was 36 months (range:1–193), while the largest number of reported cases (251; 43.4%) were children aged 1–5 years (Table 1). Almost three fourths of reported cases (429/578; 74.2%) belonged to the Roma population (Table 1). Among the Roma cases, 415 were Greek Roma (96.7%) and 14 (3.37%) were non-Greek Roma. 64 children (11.1%) had Greek origin, 67 (11.6%) were foreigners and 18 (3.1%) were refugees/immigrants residing in hosting centers.

**Table 1:** Number and percentage of measles cases by age group and origin, 8/2017-10/2018 (n = 578).

Age group (years)	(n) (%)	Roma n (%)	Greek n (%)	Foreign	Refugees/Immigrants
**< 1**	117 (20.3)	77	15	21	4
**1–5**	251 (43.4)	180	28	33	10
**6–10**	128 (22.2)	103	13	9	3
**> 10**	82 (14.3)	69	8	4	1
Total	578 (100)	429 (74.2)	64 (11.1)	67 (11.6)	18 (3.1)

**Fig 1:**
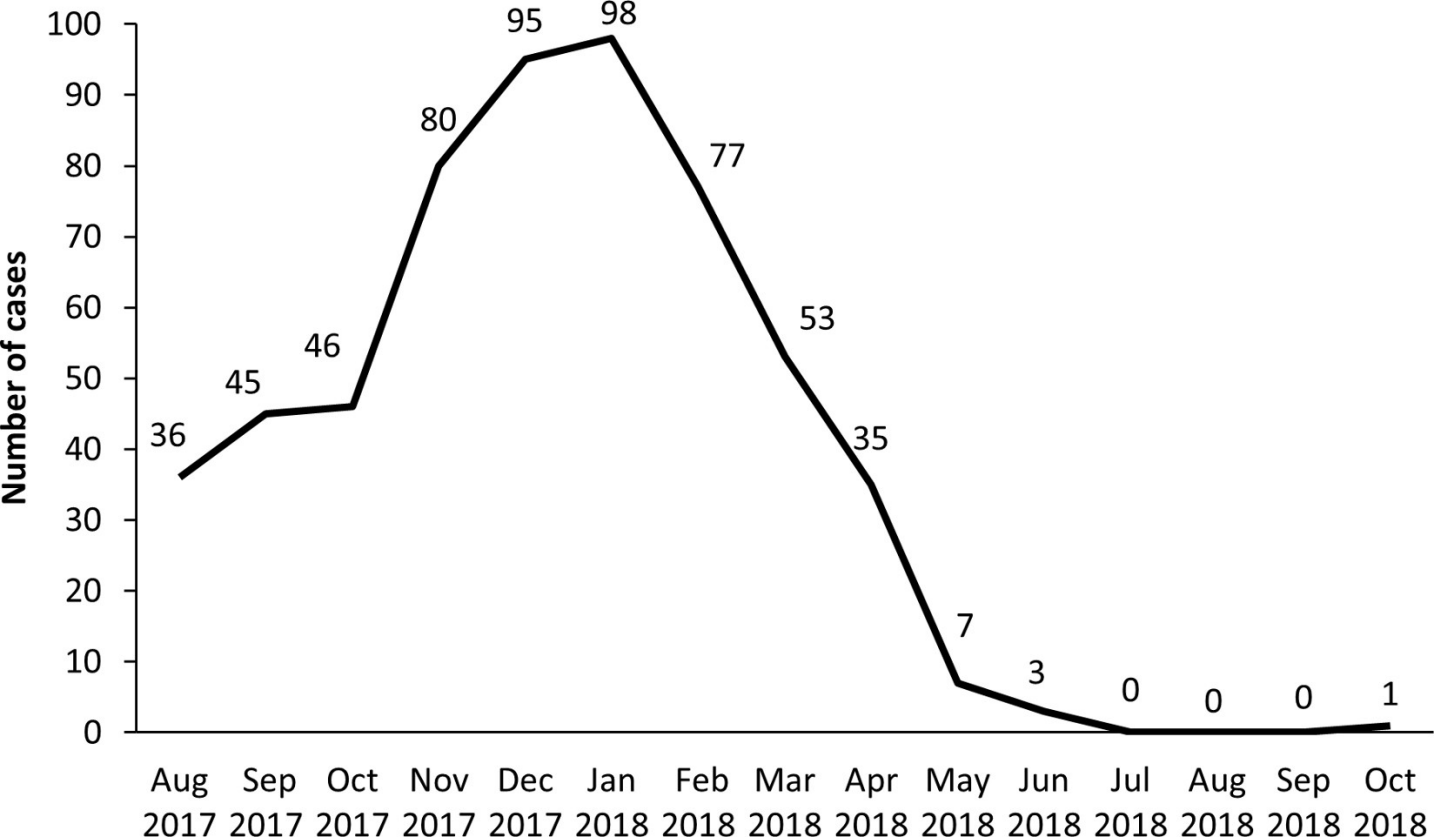
Epidemic curve of measles cases diagnosed at the major tertiary Greek Pediatric hospital, "Aghia Sofia" Children's Hospital, Athens, 08/2017-10/2018 (n = 578).

The epidemic curve (Fig 1) shows the distribution of measles cases per month. The number of children with measles diagnosed in our institution increased rapidly to peaks between November 2017 and February 2018, whereas there was reduction during autumn 2018.

Information concerning immunization status was available for 543/578 (94%) children: 497 (91.5%) were unvaccinated, 37 (6.8%) were partially vaccinated with one dose of measles containing vaccine and only 9 (1.7%) with two doses. Among the Roma cases, only 4.6% (20/429) had received one or two doses of measles vaccine, while 90.2% (387/429) were unvaccinated. Regarding children of Greek origin, 44 (68.7%) were unimmunized, 13 (20.3%) were partially vaccinated with one dose, while 6 (9.3%) had received 2 doses of measles vaccine.

The possible transmission setting was reported for 63.1% (365/578) of the cases. Transmission occurred through family member contact (291/365; 79.6%), community contact (43/365; 11.8%) or in nosocomial setting (31/365; 8.5%).

The mean (±SD) duration of symptoms before diagnosis was 4 days (±2). Most frequent symptoms and signs at presentation were: fever (570/578; 98.6%), rash (395/578; 68.3%), cough (297/578; 51%), coryza (243/578; 42%), conjunctivitis (156/578; 27%), while only 16.6% (96/578) had the pathognomonic Koplik spots.

Several children (n = 21; 3.65%) had underlying health conditions such as immunodeficiency (n = 3), histiocytosis (n = 2), diabetes mellitus type I (n = 1), IPEX syndrome (n = 1), Tay Sachs syndrome (n = 1), mental retardation (n = 2), spastic tetraparesis (n = 2), metabolic disease (n = 2), Bartter syndrome (n = 1), nephrotic syndrome (n = 1), β-thalassemia (n = 1) and Fanconi anemia (n = 1). Furthermore, 2 children were under immunosuppressive therapy (due to liver transplantation and psoriatic dermatitis respectively) and 1 child had biliary atresia.

Hospitalization was required for 342 (59.2%) children. The median age (range) of children that were hospitalized was 22.5 (1–190) months, while the median age (range) of children that did not need hospitalization was 50 (1–190) (p<0.001). Mean length (±SD) of hospital stay was 4.465 (± 3.97) days.

Laboratory findings of hospitalized children are summarized in Table 2. Hyponatremia (Νa+<135mmol/lt) was noted in 88/339 (25.9%) of children, while 13 children (3.8%) had Na+<130mmol/lt. CRP value > 20ml/lt was found in 96 (28.1%) children. 38(11.2%) of them had CRP>50mg/lt and 13(3.8%) had >100mg/lt. CRP value > 20mg/lt was found in 38% of children with complications vs 14% in children without complications (*P*<0.001).

**Table 2:** Laboratory parameters of hospitalized children diagnosed with measles in Athens 8/2017-10/2018 (n = 342).

	Reference range	Median	Min	Max	IQR
**WBC (x 10^3^/μl)**	4,000–11,000	6,000	1,380	24,200	4,500
**PLTs (x 10^3^/μl)**	150,000–450,000	259,000	37,000	2,040,000	119,000
**CRP (mg/lt)**	1–10	9.24	1	423	18.7
**Urea (mg/dl)**	10–35	18	12	55	7
**Cr (mg/dl)**	0.2–1	0.31	0.12	1.1	0.165
**SGOT (IU/lt)**	10–60	45	11	368	22
**SGPT (IU/lt)**	5–45	19	6	327	14
**Na (mmol/lt)**	135–145	136	126	145	3
**K (mmol/lt)**	3.5–5.5	4.2	2.7	5.7	0.6

Complications during the course of measles in hospitalized children are presented in Table 3. One or more complications were reported in 230 (39.8%) children. Among the most frequent complications documented were elevated transaminases (139; 40.6%), acute otitis media (72; 21%), dehydration (67; 19.6%) and pneumonia (58; 16.9%).

**Table 3:** Complications in 342 hospitalized children with measles, 8/2017-10/2018.

Complication	N	(%)
***Respiratory System***	**146**	**42.69**
ARDS	6	1.75
Upper respiratory tract involvement	7	2.05
Streptococcal tonsillitis	2	0.58
Acute otitis media	72	21.05
Pneumonia	58	16.96
Bronchiolitis	25	7.31
***Gastrointestinal system***	**148**	**43.27**
Elevated transaminases	139	40.64
Stomatitis	4	1.17
Hematemesis	5	1.46
***Central nervous system***	**19**	**5.56**
Status epilepticus	3	0.88
Altered level of consciousness	10	2.92
Encephalitis	2	0.58
Febrile seizures	4	1.17
***Hematopoietic system***	**27**	**7.89**
Neutropenia	23	6.73
Thrombocytopenia	2	0.58
Anemia	2	0.58
***Cardiac system***	**5**	**1.46**
Arrythmia	1	0.29
Myocarditis	3	0.88
Kawasaki disease	1	0.29
***Other***	**85**	**24.86**
Dehydration	67	19.59
Skin infection	9	2.63
Hematuria	1	0.29
Sepsis	7	2.05
Jaundice	1	0.29

Complications were more common in children ≤3 years than in older children (>3years) (*P*<0.001). Younger children (≤3years) were more susceptible to respiratory system involvement (*P*<0.001), acute otitis media (*P*<0.05), bronchiolitis (*P*<0.001), neutropenia (*P*<0.05) and elevated liver enzymes (*P*<0.001), whereas older children had usually presented with dehydration (*P*<0.05) and thrombocytopenia (*P*<0.05). Among children with sepsis (n = 7, (2.05%), 2 had *S*. *pneumoniae* bacteremia.

Eleven children (3.2%) required intensive care admission for altered mental status/status epilepticus (3), sepsis (2) and ARDS (6). The mean (±SD) duration of stay in the Pediatric Intensive Care Unit (PICU) was 4 days (±2.8). Two of the 11 children had underlying health conditions, namely Tay-Sachs syndrome (1) and dystrophy/possible metabolic disease (1), whereas the 9 remaining children were previously healthy. Among these children, one death was reported, concerning an 11-month old unvaccinated infant, with underlying dystrophy, who died of sepsis.

Regarding treatment, 119/342 (34.8%) children with measles were treated with antibiotics because of possible or confirmed bacterial coinfection. More specifically, 98 (28.7%) children were treated with a single antibiotic, 17 (5%) with two antibiotics and 4 (1.2%) with three antibiotics. The most frequently used antibiotics were ampicillin/b lactamase inhibitor (37) and 3rd generation cephalosporin (35). Six (1.75%) children with gastrointestinal involvement (hematemesis, severe vomiting) were treated with PPIs, whereas 47 (13.7%) children with respiratory system involvement received nebulized bronchodilators. Acyclovir was administered to one child with clinical presentation of encephalitis, 10 (2.9%) children were treated with ribavirin,19 (5.5%) received intravenous immune globulin (IVIG). and 18 children (5.3%) Vitamin A. Three children (0.87%) with hemodynamic instability and reduced ejection fraction on cardiac ultrasonography received inotropic agents (dobutamine, dopamine, noradrenaline). All children who admitted to the PICU were administered IVIG and most of them received vitamin A and ribavirin (10 (90.9%) and 8 (72.7%) respectively).

## Discussion

In the present study we presented a significant number of children who were diagnosed with measles during the Greek measles epidemic that happened between 2017–2018. Our data confirm that measles continues to be an important public health problem and especially during epidemics causes significant morbidity and complications.

Some delay in diagnosis of the early measles cases could be explained by the fact that younger pediatricians were not familiar and might not have considered measles as a possible diagnosis at the outbreak onset. Similar challenges had occurred in recent measles outbreaks in other countries [10, 11]. In addition, delays could be explained by the fact that several children did not present with the classic triad of symptoms and were at the beginning managed to a different direction [4, 12, 13].

No significant difference in the prevalence of measles between the genders was noted, which was also described in other European studies [11, 12, 14]. The greatest prevalence was noted in the 1–5 years age group (43.4%), followed by the infants under 1 year of age (20.3%). Similar findings regarding the most susceptible age groups were described in other recent European studies [11, 15, 16]. The high vulnerability of infants and toddlers could be related to the actual immunization policy, as the first measles vaccine dose was recommended in Greece at the age of 12–15 months and the 2^nd^ dose at 4 years. This could be associated with an immunity gap in infants between 6–12 months. The weaning immunity in women of childbearing age could be another reason that leads to a gap in protective immunity, as it does not allow the indirect protection of infants through placental transferred maternal antibodies [17].

Regarding the vulnerability of the 1-5-year age group, there is always the possibility of failure to develop a satisfactory immune response after the first dose of measles vaccine. Although in Greece measles vaccination coverage with the first dose exceeds 95%, there was a recent reduction in coverage, a fact that enlarges the reservoir of susceptible children under 5 years of age [18, 19]. In addition, coverage with the second dose is under 90%, lower than the WHO target of 95% that is a precondition to herd immunity [3, 4].

Most children who developed measles in our study were unvaccinated or partially vaccinated as also reported in previous measles epidemics in Greece [5, 20]. These results are similar to results described in other European studies experienced large outbreaks at the same period [11, 21, 22]. A number of measles cases in fully vaccinated children were also reported (1.7%), which might be attributed to vaccine failure [4, 23, 24].

The outbreak mainly affected the minority of Roma (74.2%), the vast majority of whom were unvaccinated (90.2%), which was noted in national studies in Greece and Spain [4, 25]. According to the last national immunization study in Greece only 8% of Roma children have received 2 doses of the measles vaccine [19]. For these reasons, there was a modification of Greek National immunization schedule during the epidemic that recommended 1^st^ dose of MMR vaccine at 12 months and 2^nd^ dose at 15 months that may be affected the epidemic curve [4].

Roma population faces several challenges regarding their access to immunization including accessible medical services, language barriers, illiteracy, discrimination, cultural beliefs [8].

To this direction, mobile vaccination vans, door -to-door knocking, school or community-based immunization programs and translator services would be very effective in increasing vaccination uptake—and access to health care in general- in this group [8, 26].

The low number of measles cases in refugees/immigrants demonstrates the success of Greek refugee vaccination project that started before the propagation of the epidemic in Greece low (Programme PHILOS) [27]. However, as incoming immigrants are continually accepted in hosting centers, constant awareness is needed for rapid response.

Regarding the probable transmission setting, the majority of the cases occurred within families and community, findings that are similar to other studies [10]. However, a significant nosocomial transmission was detected, as children presented at hospitals at an early stage, without a rash or with atypical symptoms and they were not identified and isolated early leading to secondary cases, including unvaccinated Health Care Workers (HCWs) [12, 25]. HCWs affected by measles are thought to be a major challenge for the containment of the epidemic. According to the Greek surveillance data until May 2018, 117 measles cases among HCWs had been identified, of whom 59.6% were unvaccinated and 30.3% were partially vaccinated [28].

In the present study a high hospitalization rate of 59.2% was found, but rates lower than 20%, or higher than 60% have also been reported [11, 12, 17, 25, 29, 30]. This wide range of hospitalization rates worldwide may be related to differences in management and surveillance systems, health policies and the affected age groups. The relatively high hospitalization rate in our study may possibly reflect under-reporting of mild cases or that children with a higher risk of complications and with more severe symptoms were more likely to seek medical care [4, 31].

Regarding laboratory parameters of hospitalized children with measles, there was a significant number of children with mild (26%) or severe (3.8%) hyponatremia at presentation, a finding that is not regularly described. Elevated transaminases were detected in 40.6% of measles cases, which was also found in other studies [29, 31].

Elevated CRP (>20mg/L) was associated increased risk of developing complications, a finding that was also described in a study in BMJ in 2019 [32]. Although the impact of bacterial coinfection on CRP value is unknown, point-of-care CRP testing might be a significant tool to help pediatricians in the identification of children requiring hospital management or antibiotic administration. Almost one third of children with measles in our study were treated with antibiotics, however higher use of antimicrobials have been described in other studies (73%-96,8%) [29, 31]. Antibiotic consumption should be taken into account when assessing the indirect cost of measles and the benefits of immunization, as described in a recent metanalysis [33].

Additional therapeutic interventions like ribavirin, IVIG or Vitamin A were used in specific cases. All children who admitted in the Intensive Care Unit (ICU) were given IVIG and most of them also received vitamin A and ribavirin. Variable use of vitamin A in measles cases has been reported in different studies despite official recommendations [2, 29, 34, 35].

In our setting, a significant number of children with measles developed complications (39.8%). These results are similar to those described in other European countries which experienced large measles outbreaks in the same time period [12, 17, 29, 31]. Complications were more common in younger children especially regarding respiratory system involvement, acute otitis media, bronchiolitis or neutropenia, whereas older children usually presented with dehydration and thrombocytopenia. Different results regarding complication rates by age group have been described depending on the country [16, 17, 31].

There was need for PICU admission in 1.9% of children due to severe complications, such as ARDS, sepsis and altered mental status and there was one death. Need for ICU hospitalization and deaths especially in children with underlying diseases have also been described in other studies in developed countries [2, 29, 36].

High hospitalization rates and complications associated with measles outbreak are substantial and incur high financial costs for public health systems, as described in several European studies [37–39].

As many countries are trying to cope with the SARS-COV-2 pandemic, immunization rates are falling and several countries have suspended measles vaccination campaigns [40, 41]. This situation would probably increase unimmunized populations and lead to increased risk for future measles outbreaks [41].

Limitations of the present study include that this is a single center retrospective study and do not reflect the whole measles epidemic in Greece. However, it represents a significant number of pediatric cases (26%) and presents clinical and laboratory characteristics that are not included in the national database. In addition, some of the measles cases immunization data, especially in Roma population, were self-reported and could not be confirmed from immunization booklets. However, it is unlikely that this possible bias changed our results.

## Conclusion

During the 2017–2018 measles outbreak in Greece, it was noted that measles is still not an innocent viral infection, as it is characterized by high morbidity rates and complications. Pockets of unvaccinated populations could be the source of new outbreaks, resulting in significant cost in public health. To address future challenges, high measles vaccination coverage should be achieved, as well as closing immunity gaps in the population and ensuring high-quality laboratory surveillance.

## Supporting information

S1 Data (DOCX)

S2 Data (XLSX)
