# Supplementary material for: Measles epidemic in pediatric population in Greece during 2017–2018: Epidemiological, clinical characteristics and outcomes
Source: PLoS One. 2021 Jan 20;16(1):e0245512. doi: 10.1371/journal.pone.0245512 (PMC7817010; doi:10.1371/journal.pone.0245512)
Supplement: S1 Data — (DOCX) [file pone.0245512.s001.docx]

|  | | Frequency | Percent | Cumulative Percent |
| --- | --- | --- | --- | --- |
| Valid | August 2017 | 36 | 6,25 | 6,25 |
|  | September 2017 | 45 | 7,81 | 14,06 |
|  | October 2017 | 46 | 7,99 | 22,05 |
|  | November 2017 | 80 | 13,89 | 35,94 |
|  | December 2017 | 95 | 16,49 | 52,43 |
|  | January 2018 | 98 | 17,01 | 69,44 |
|  | February 2018 | 77 | 13,37 | 82,81 |
|  | March 2018 | 53 | 9,20 | 92,01 |
|  | April 2018 | 35 | 6,08 | 98,09 |
|  | May 2018 | 7 | 1,22 | 99,31 |
|  | June 2018 | 3 | 0,52 | 99,83 |
|  | October 2018 | 1 | 0,17 | 100,0 |
| Total | | 578 | 100,0 |  |
|  | |  |  |  |
